# A single center case series of immune checkpoint inhibitor-induced type 1 diabetes mellitus, patterns of disease onset and long-term clinical outcome

**DOI:** 10.3389/fimmu.2023.1229823

**Published:** 2023-08-21

**Authors:** John Marsiglio, Jordan P. McPherson, Magdalena Kovacsovics-Bankowski, Joanne Jeter, Christos Vaklavas, Umang Swami, Douglas Grossmann, Alyssa Erickson-Wayman, Heloisa P. Soares, Katie Kerrigan, Berit Gibson, Jennifer Anne Doherty, John Hyngstrom, Sheetal Hardikar, Siwen Hu-Lieskovan

**Affiliations:** ^1^ Department of Internal Medicine, University of Utah Health, Salt Lake City, UT, United States; ^2^ Huntsman Cancer Institute, University of Utah Health, Salt Lake City, UT, United States; ^3^ Department of Dermatology, University of Utah Health, Salt Lake City, UT, United States; ^4^ Department of Population Health Sciences, University of Utah Health, Salt Lake City, UT, United States

**Keywords:** immune checkpoint inhibitors, immunotherapy, immune-related adverse events, type 1 diabetes, proteomics

## Abstract

**Background:**

Type 1 diabetes mellitus (T1DM) is a rare, but serious immune-related adverse event (irAE) of immune checkpoint inhibitors (ICIs). Our goal was to characterize treatment outcomes associated with ICI-induced T1DM through analysis of clinical, immunological and proteomic data.

**Methods:**

This was a single-center case series of patients with solid tumors who received ICIs and subsequently had a new diagnosis of T1DM. ICD codes and C-peptide levels were used to identify patients for chart review to confirm ICI-induced T1DM. Baseline blood specimens were studied for proteomic and immunophenotypic changes.

**Results:**

Between 2011 and 2023, 18 of 3744 patients treated at Huntsman Cancer Institute with ICIs were confirmed to have ICI-induced T1DM (0.48%). Eleven of the 18 patients received anti-PD1 monotherapy, 4 received anti-PD1 plus chemotherapy or targeted therapy, and 3 received ipilimumab plus nivolumab. The mean time to onset was 218 days (range 22-418 days). Patients had sudden elevated serum glucose within 2-3 weeks prior to diagnosis. Sixteen (89%) presented with diabetic ketoacidosis. Three of 12 patients had positive T1DM-associated autoantibodies. All patients with T1DM became insulin-dependent through follow-up. At median follow-up of 21.9 months (range 8.4-82.4), no patients in the melanoma group had progressed or died from disease. In the melanoma group, best responses were 2 complete response and 2 partial response while on active treatment; none in the adjuvant group had disease recurrence. Proteomic analysis of baseline blood suggested low inflammatory (IL-6, OSMR) markers and high metabolic (GLO1, DXCR) markers in ICI-induced T1DM cohort.

**Conclusions:**

Our case series demonstrates rapid onset and irreversibility of ICI-induced T1DM. Melanoma patients with ICI-induced T1DM display excellent clinical response and survival. Limited proteomic data also suggested a unique proteomic profile. Our study helps clinicians to understand the unique clinical presentation and long-term outcomes of this rare irAE for best clinical management.

## Introduction

The use of immune checkpoint inhibitors (ICIs) has been increasingly prevalent in cancer treatment. ICIs target immune checkpoint molecules that regulate immune cell activation, such as cytotoxic T lymphocyte associated antigen-4 (CTLA-4) and programmed cell death-1 (PD-1). The binding of CD80/86 to CTLA-4 and PD-1/PD-L1 normally inactivates T-cells and blocks pro-apoptotic pathways, eventually leading to effector T cell exhaustion (1). By overexpressing these ligands, cancer cells block T cell activation and evade the immune system. ICIs block these interactions and allow for immune overactivation to take place. Unfortunately, this same mechanism also blocks the normal negative signaling of T cells, leading to autoimmune-like toxicities known as immune-related adverse events (irAEs) (1).

irAEs can affect nearly every organ system and be life-threatening. While most irAEs are reversible with steroid treatment, some are irreversible. Endocrinopathies account for 4-30% of reported irAEs and are usually permanent (1). The most common immune-mediated endocrinopathy is hypothyroidism, which is present in about 10% of patients treated with anti-PD-1 monotherapy, but easily treatable (1). In contrast, ICI-induced type 1 diabetes mellitus (T1DM) is rare, but associated with significant long-term morbidity.

The mechanism of ICI-induced T1DM is not fully understood. Both autoimmune and ICI-induced T1DM are thought to be caused when autoreactive CD4+ T cells, CD8+ T cells, and B lymphocytes destroy the insulin-producing beta cells in the islets of Langerhans. ICI-induced T1DM is only sometimes associated with positive T1DM-associated autoantibodies, suggesting a unique pathophysiology (2). Common islet cell protein targets of these autoantibodies include glutamic acid decarboxylase (GAD), insulin (IA), insulinoma associated antigen-2 (IAA), zinc transporter 8 (ZnT8), and islet cells (ICA). Given the serious and irreversible nature of ICI-induced T1DM, it is critical to identify biomarkers to predict risk of this rare complication. While genetic associations with human leukocyte antigens (HLA) have been documented in autoimmune T1DM, HLAs specific for irAEs are an exciting new field (3, 4). Predictive biomarkers of ICI-induced T1DM have also been proposed but are not yet fully understood (5).

Despite several published cohorts and case reports of ICI-induced T1DM, impact on treatment outcomes remains unclear given the small number of patients reported thus far (2, 6–12). About half of the patients in these cohorts were treated for melanoma. Nearly all received anti-PD1 or PD-L1 therapy for active treatment in stage IV disease as well as adjuvant therapy in other stages. The clinical course in these cohorts is well described. On average, the time to T1DM diagnosis is approximately 23 weeks, 60% of patients present in diabetic ketoacidosis, 40% of patients have positive autoantibodies, and 80% of patients have low or undetectable c-peptide (2, 6–12). However, treatment response data is reported for only a few studies, with variable response rates. Four cohorts reported positive treatment response data (7–10). These cohorts showed objective response rates (ORR) of up to 80-70% in patients with melanoma although results varied (7–10). These data suggest greater radiographic clinical response in melanoma patients with ICI-induced T1DM compared to historical controls (reported ORR 30-50%). To better characterize clinical outcomes, we reviewed all cases of ICI-induced T1DM at our institution. Here we report clinical and proteomic data for this large cohort of patients after long-term follow-up.

## Materials and methods

This was a single-center case series of adult patients with solid tumors at Huntsman Cancer Institute at the University of Utah Health. Electronic medical records were searched for patients who received ICIs (ipilimumab, pembrolizumab, nivolumab, cemiplimab, atezolizumab, durvalumab and avelumab) during an 12-year period (2011 to 2023). To identify T1DM diagnoses after ICI therapy, the enterprise data warehouse (EDW) at the University of Utah used ICD codes for T1DM (ICD-10-CM E10 and ICD-9-CM 250.01) or obtained C-peptide levels measured after starting ICI therapy. A chart review of these cases was then conducted to confirm diagnosis of ICI-induced T1DM.

Chart review confirmed ICI-induced T1DM cases if they met the following inclusion criteria: sustained insulin dependence with a low or undetectable C-peptide level through longitudinal follow-up after high dose steroids were stopped if they were started. Patients were excluded due to the following reasons: 1) pre-existing T1DM diagnosis, 2) C-peptide drawn for other reasons in non-diabetic patient, 3) non-insulin-dependent worsening of preexisting T2DM, 4) new T2DM diagnosis, 5) worsened hyperglycemia related to steroid use and 6) insufficient data.

Blood chemistry and complete blood count prior to ICI therapy, at six weeks prior to T1DM development, and three weeks prior to T1DM development were available for 11 of 18 patients. Lab data was analyzed by two-tailed, two-sample t-tests for significant changes from baseline to investigate possible association with the development of T1DM.

Through an IRB-approved protocol, peripheral blood was collected from patients with melanoma prior to starting immunotherapy (baseline levels). Plasma was then isolated and stored at –80C. Baseline plasma from 2 patients who later developed ICI induced T1DM and 5 patients who did not have any irAE were available for study and sent for a multiplexed Proximity Extension Assay (PEA), developed by Olink Proteomics (Uppsala, Sweden). This assay was used to measure protein abundance directly in the plasma obtained from patients. In brief, the PEA is a dual recognition assay, where each protein is recognized by oligonucleotide-labeled antibody probes, then amplified and quantified by PCR. The baseline abundance levels of 1472 plasma proteins were analyzed using 4 pre-defined PEA-panels (Olink® Multiplex Inflammation, Oncology, Cardiometabolic and Neurology). The results were analyzed using the Olink® proteomics program that can be found at https://olink.com/products-services/data-analysis-products/olink-statistical-analysis-app/.

## Results

### Patient demographics

**Figure 1:**
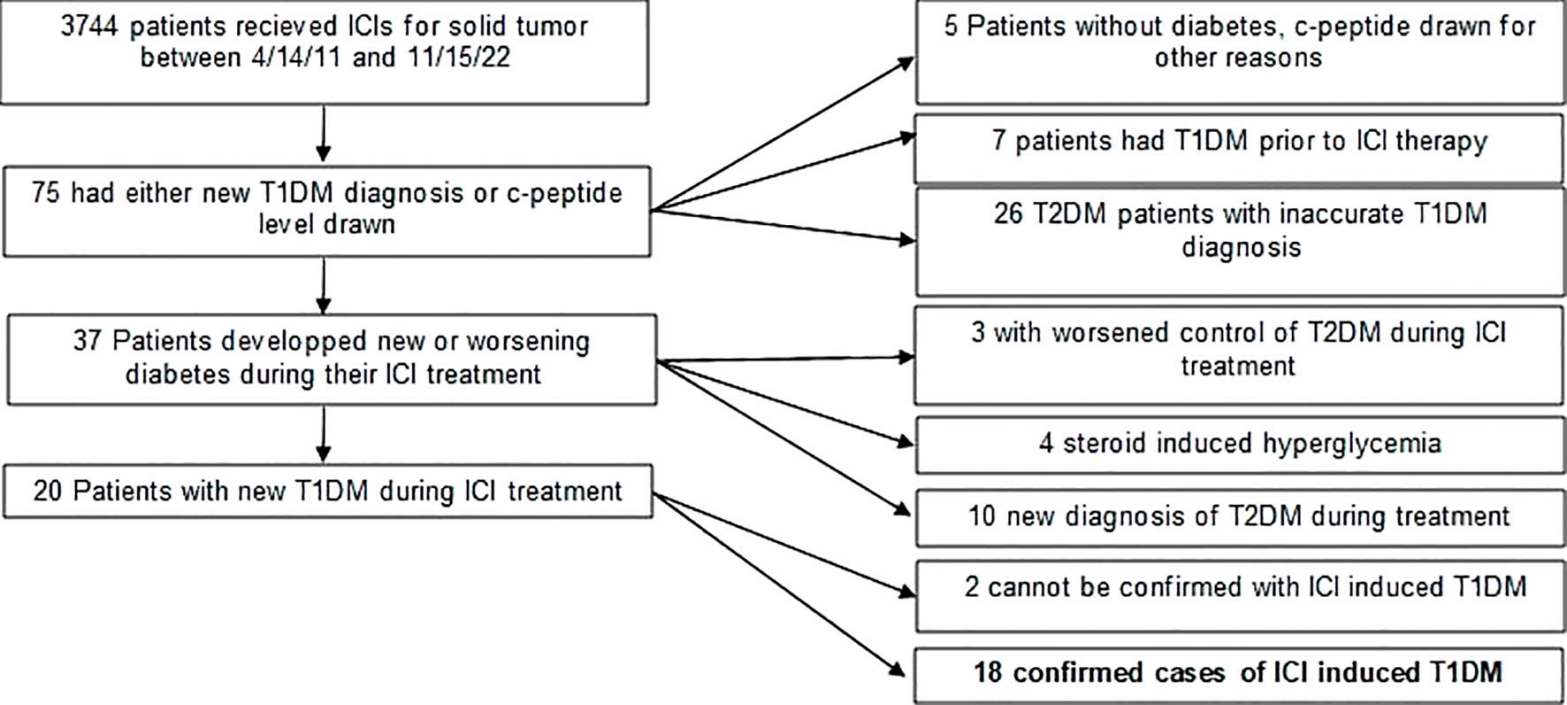
Methods flowchart for identifying patients.

**Figure 2:**
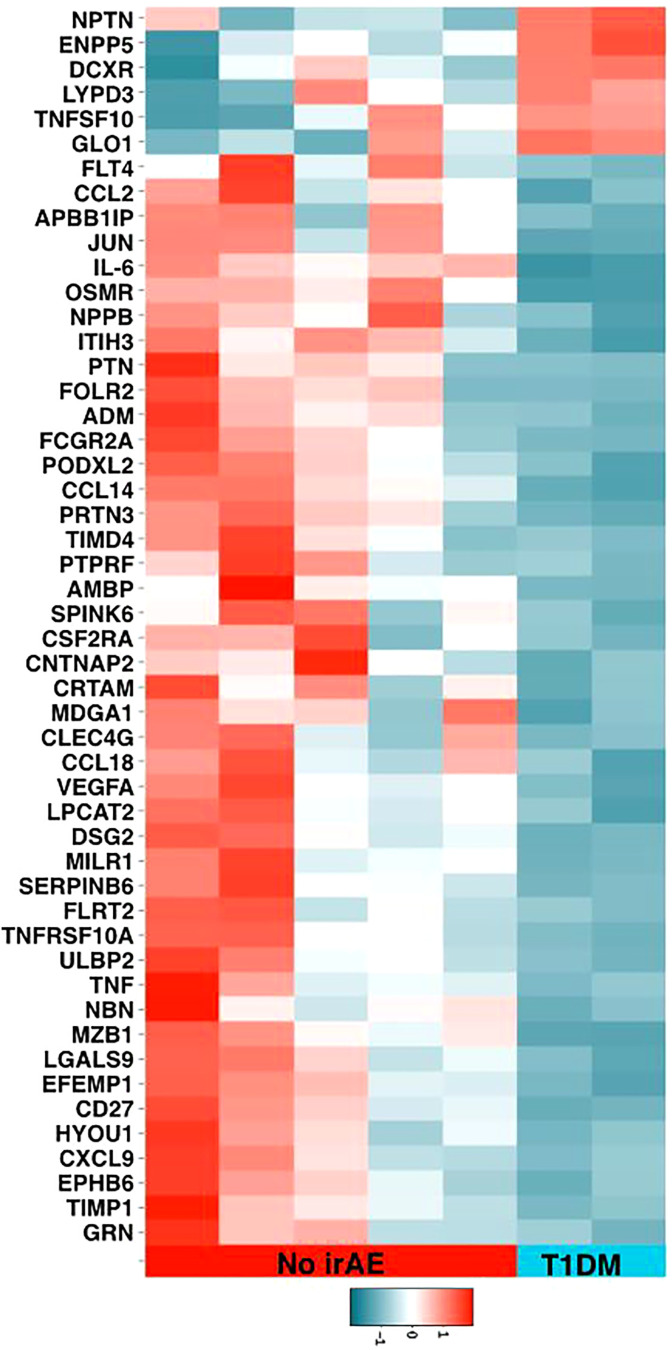
Olink® Proteomic data heatmap.

Between April 14, 2011, and April 1, 2023, 75 of 3744 patients who received ICIs at HCI had a T1DM ICD-10 diagnosis or C-peptide levels measured after the first cycle (**Figure 1**). Of these, 23 (35%) were identified as having type II diabetes without use of insulin, and five (7%) had C-peptide levels not indicative of insulin dependence. Seven (9%) had a documented T1DM diagnosis prior to ICI treatment, ten (13%) developed non-insulin dependent T2DM during treatment, seven (9%) had worsening of T2DM during ICI treatment leading to a nonpermanent insulin requirement, and two patients (3%) did not have sufficient data to confirm a T1DM diagnosis outside of their listed diagnosis. Eighteen patients were confirmed to have ICI-induced T1DM with a prevalence of 0.48% and are summarized in **Table 1**. There were eleven males and seven females. The mean age at the time of treatment was 61 years (range 32-75). All patients were diagnosed between 2012-2022. None of these patients had a history of diabetes prior to starting ICIs (**Figure 2**).

**Table 1:** Baseline clinical characteristics of patients prior to development of immune checkpoint inhibitor-induced diabetes.

#	Age/Sex	Race	Disease	BRAF Status	Stage Prior to Treatment	Treatment Type	Prior Treatment History	Treatment
1	68M	Caucasian	Lentigo maligna melanoma	Wild Type	Stage IV M1b	Active	Resection	Nivolumab 480 mg
2	51M	Caucasian	Nodular melanoma	V600E	Stage IIIB	Adjuvant	Seviprotimut-L clinical trial	Pembrolizumab 200 mg
3	32M	Caucasian	Nevoid melanoma	Wild Type	Stage IIIA	Adjuvant	Resection	Pembrolizumab 200 mg
4	37M	Caucasian	Melanoma	V600E	Stage IV M1d	Active	None	Nivolumab + ipilimumab
5	66M	Caucasian	Melanoma	V600E	Stage IIIA	Adjuvant	Resection	Nivolumab 480 mg
6	56M	Caucasian	Nodular melanoma	V600E	Stage IV M1b	Adjuvant	Adjuvant ipilimumab 2 cycles with G3 colitis	Pembrolizumab 200 mg
7	63F	Caucasian	Acral melanoma	Wild Type	Stage IIIB	Adjuvant	Resection	Pembrolizumab 200 mg
8	67F	Caucasian	CCA	NA	Stage IV	Active	Chemotherapy/radiation/brachytherapy	Pembrolizumab 200 mg + FOLFOX
9	55M	Caucasian	Prostate cancer	NA	Stage IV	Active	FOLFOX, degarelix, bicalutamide, enzalutamide, abiraterone	Pembrolizumab 200 mg+ enzalutamide
10	70F	Pacific Islander	Papillary RCC type 1	NA	Stage IV	Active	Resection, sunitinib	Nivolumab 480mg
11	67M	Caucasian	Invasive SCC of tongue	NA	Stage IV	Active	Resection, cisplatin	Pembrolizumab 200mg
12	60F	Caucasian	Metastatic CCA	NA	Stage IV	Active	Abraxane + gemcitabine + cisplatin	Nal-IRI/5-FU/Nivolumab
13	57F	Caucasian	Breast carcinoma (ER/PR+, HER2 unamplified)	NA	Stage IIA	Neoadjuvant	none	Cemiplimab, paclitaxel, REGN3767
14	71F	Caucasian	Clear cell renal cell carcinoma	NA	Stage IV	Active	nephrectomy	Nivolumab + ipilimumab
15	65M	Caucasian	HPV mediated SCC of oropharynx	NA	Stage IV	Active	Resection, radiation, cisplatin	Pembrolizumab 400mg
16	75M	Caucasian	Melanoma	Wild Type	Stage IV	Active	Resection	Pembrolizumab 200mg +TVEC
17	72F	Caucasian	Mucosal Melanoma	Wild Type	Stage IV	Active	Resection, radiation therapy	Ipilimumab 1mg/kg + nivolumab 3mg/kg
18	69M	Caucasian	Acral Melanoma	Wild Type	Stage IIID	Adjuvant	Resection	Pembrolizumab 400mg

NA, not available.

Malignancies included 10 melanomas (1 lentigo maligna, 2 nodular, 1 nevoid, 2 acral, 1 mucosal and 3 unspecified), 2 cholangiocarcinomas, 1 prostate cancer, 1 papillary renal cell carcinoma, 1 clear cell renal carcinoma, 2 head and neck squamous cell carcinomas, and 1 invasive breast ductal carcinoma (**Table 1**). In the melanoma group, 4 of 10 patients had a BRAF V600E mutation, 2 were stage IIIA, 2 were stage IIIB, 1 was stage IIID and 5 were stage IV at the time of initiating ICIs. Of the patients with other malignancies, 7 were stage IV, while one was stage IIA on neoadjuvant therapy. Treatment history prior to ICIs varied widely among the patients. Six melanoma patients had prior surgery, one prior surgery and radiation, and 5 of 6 started ICIs as adjuvant therapy. The sixth had recurrence and subsequently started therapy for unresectable disease. One patient was previously treated with ipilimumab that was later stopped, 1 was on a seviprotimul-L clinical trial, and 1 had no prior treatment. Of the non-melanoma patients, prior treatments were more diverse, as shown in **Table 2**. Two had prior chemotherapy/radiation, 3 had prior chemotherapy, 1 had radiation therapy and 2 were treated with kinase inhibitors. Only 3 of the non-melanoma patients had prior surgical resections. Ten of 16 patients received anti-PD1 monotherapy (8 pembrolizumab, 3 nivolumab), 2 received pembrolizumab plus chemotherapy (FOLFOX) or enzalutamide, 1 received nivolumab plus chemotherapy (nanoliposomal irinotecan/fluorouracil), 1 received cemiplimab, anti-LAG-3 plus chemotherapy (REGN3767, an anti-lymphocyte activation gene 3 protein, LAG-3 + paclitaxel), 1 received pembrolizumab and TVEC and 3 received ipilimumab plus nivolumab. Of the melanoma patients, 4 received active treatment, while the other 6 received adjuvant treatment. In the other cancers group, 7 received active treatment, while 1 received neoadjuvant treatment.

**Table 2:** Clinical outcomes of patients with immune checkpoint inhibitor-induced diabetes.

#	Cycles Prior to T1DM	Total Cycles Given	Days to T1DM Onset	Other Endocrine irAEs	Other irAEs	Best Tumor Response	ICI Continued After Clinical Improvement of T1DM	OS (months)	PFS (months)
1	6	16	172	None	G1 hypopigmentation, G2 arthralgias	CR	Yes	NR	NR
2	9	9	195	G2 hypothyroidism	G2 anorexia, G1 dysgeusia	NED	No	NR	NR
3	11	11	315	G2 hyperthyroidism, G2 hypothyroidism	G1 joint stiffness, G1 elevated AST/ALT	NED	No	NR	NR
4	11	15	418	None	G3 colitis treated with steroids, infliximab	PR	Yes	25.5	NR
5	9	9	363	None	G3 pancreatitis	NED	No	NR	NR
6	3	18	71	None	G1 arthralgias	NED	Yes	NR	NR
7	13	13	280	None	NA	NED	No	NR	NR
8	8	8	131	None	G1 fatigue, nausea, vomiting	PR	No	13.8	2.4
9	NA	NA	119	G2 hypothyroidism	NA	PD	No	22.9	2.7
10	11	13	374	G2 adrenal insufficiency	NA	SD	No	NR	2.8
11	1	4	23	colitis, ungraded	NA	PD	Yes	18.6	12.4
12	2	2	392	G2 hypothyroidism	G1 diarrhea, G1 sore throat, G1 mouth sores	PD	No	18.9	13.7
13	2	2	215	None	G3 rash, G2 dry mouth, G1 hoarseness	CR	No	NR	NR
14	2	2	36	None	G1 rash	PD	No	NR	2.14
15	3	3	129	None	NA	SD	No	NR	8
16	18	18	367	None	G2 neuropathy	CR	No	NR	NR
17	5	9	68	G2 hypothyroidism	G1 rash, G1 diarrhea	PR	Yes	NR	NR
18	6	11	252	G2 hypothyroidism	G1 rash, G1 dry mouth	NED	Yes	NR	NR

G, grade; CR, complete response; PR, partial response; SD, stable disease; PD, progression of disease; NED, no evidence of disease progression; OS, overall survival; PFS, progression free survival; NR, not reached; NA, not available.

### Onset of ICI-induced T1DM

**Figure 3:**
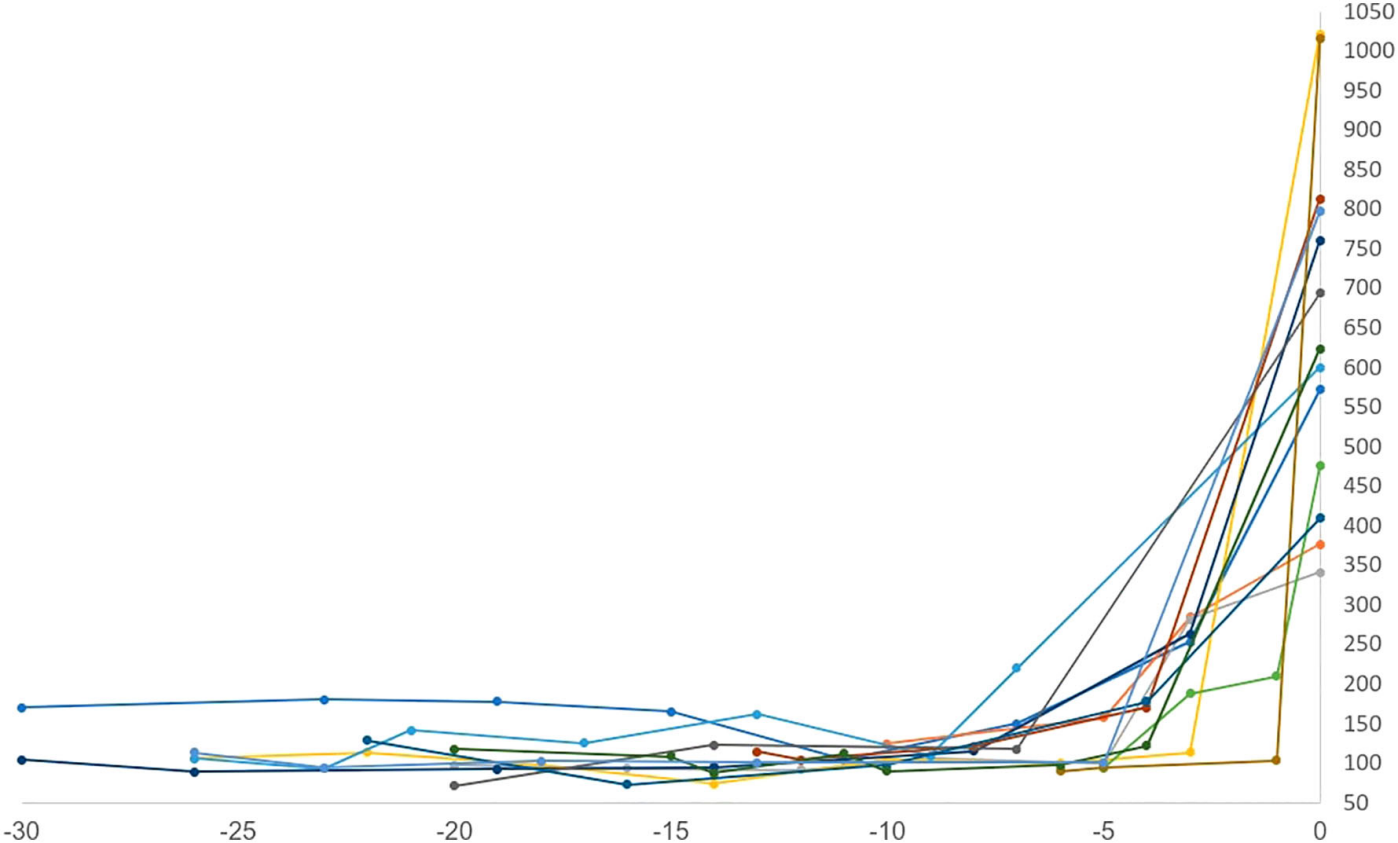
Time to onset (weeks) vs random serum glucose (mg/dl). Onset of hyperglycemia was rapid and severe for some patients.

Sixteen patients (88%) had diabetic ketoacidosis at presentation (**Table 3**). Mean hemoglobin A1c on presented was 8.8 (range 6-11.4%), and glucose was 633 mg/dl (range 341-1021). The mean time to T1DM onset was 218 days (median 205, range 23-418 days) from ICI initiation. Median number of cycles completed prior to diagnosis was 6 (range 1-18). Interestingly onset of hyperglycemia was rapid and severe in some patients (**Figure 3**). Compared to baseline, glucose was usually significantly elevated at 3 weeks prior to diagnosis but not significantly different 6 weeks prior. Fifteen of 16 DKA patients had an elevated anion gap (mean 19, range 15-27). Serum bicarbonate was low in these patients (mean 13.6 mEq/l, range 5-24mEq/l). Urinary ketones were positive on all 11 of the patients checked. C-peptide was checked at this time in 12 patients; it was undetectable in 4 patients, low in 5, and normal in 3. Lipase levels were checked in 3 patients and elevated in one who had prior grade 3 pancreatitis as an irAE. Only 2 patients did not present with DKA, but both had elevated hemoglobin A1c (8.4 and 9.2) as well as elevated blood glucose (376 and 760) consistent with the rest of our cohort (**Table 3**). One of these 2 patients had an undetectable C-peptide, while the other had a low level when tested. All patients required insulin therapy through the duration of follow-up.

**Table 3:** Clinical presentation at time of initial identification of immune checkpoint inhibitor-induced type 1 diabetetps.

#	DKA	A1C (%)	Peak Blood Glucose (mg/dL)	Bicarbonate (mEq/L)	Anion Gap (mEq/L)	Urine Ketones	C-peptide (ng/mL)	Lipase (U/L)	Autoantibodies (+/-)
1	Yes	8.1	572	22	16	Positive	NA	NA	NA
2	No	8.4	376	20	14	NA	0.2	NA	NA
3	Yes	7.7	341	24	18	Positive	0.4	12	GAD-, IA2-, insulin-, ZnT8-
4	Yes	10.3	1021	20	17	Positive	NA	NA	NA
5	Yes	11.4	600	8	21	Positive	<0.1	347	NA
6	No	9.2	760	21	12	NA	<0.1	43	GAD-, IA2-, ICA-, ZnT8-
7	Yes	10.4	459	9	10	Positive	0.5	NA	GAD-, IA2-, ICA-, ZnT8-
8	Yes	8.7	475	5	26	Positive	NA	NA	GAD+, IA2-, ICA-, ZnT8-
10	Yes	11.2	813	6.5	17	NA	NA	50	IA2-, GAD-
11	Yes	8.0	694	NA	17	NA	<0.1	NA	GAD-, IA2-, ICA-, ZnT8-
12	Yes	6.0	NA	NA	NA	NA	NA	NA	NA
13	Yes	7.6	767	5	27	Positive	0.2	NA	GAD+, IA-2-, insulin-, ICC-, ZnT8+
14	Yes	10.6	475	8	17	NA	0.5	NA	GAD-, insulin-
15	Yes	6.8	1015	6	35	Positive	0.4	NA	GAD+, IA-2-, insulin-, ZnT8-
16	Yes	8.3	409	19	18	Positive	<0.1	NA	GAD-, IA-2-
17	Yes	8.5	623	18	15	Positive	0.3	NA	GAD-, IA2-, ICA-, ZnT8-
18	Yes	8.4	797	14	19	Positive	<0.1	NA	GAD-, IA2-, ICA-, ZnT8-

NA, not available.

### Autoantibodies and ICI-induced T1DM

Autoantibodies were tested in a total of 12 patients as shown in **Table 2**. Tested autoantibodies included anti-glutamic acid decarboxylase (anti-GAD), anti-insulin (IA), anti-insulinoma associated antigen-2 (IAA), anti-zinc transporter 8 (ZnT8), and anti-islet cells (ICA). Two had elevated anti-GAD, and another had both elevated anti-GAD and anti-ZnT8. Autoantibody status prior to T1DM diagnosis was not available for all patients. All patients presented in DKA.

### Other irAEs

Eight of the 18 patients experienced other endocrine irAEs. Seven of them experienced hypothyroidism requiring replacement medication, with 1 patient initially experiencing hyperthyroidism requiring medication (grade 2). One experienced grade 2 adrenal insufficiency. Eleven patients experienced a wide range of other irAEs that are shown in **Table 2**. Most of the experienced irAEs were grade 1 or 2, and common including liver enzyme elevations, fatigue, and arthralgias. Severe irAEs (grade 3 or greater) occurred in three of those 10 patients, 1 had grade 3 colitis, 1 had grade 3 pancreatitis, and 1 had a grade 3 rash. All required steroids and inpatient treatment. Of these 3 patients, 2 stopped their ICIs due to these other severe irAEs while 1 patient continued pembrolizumab after grade 3 colitis resolved. In total, ICIs were continued after the diagnosis of T1DM in six of the 18 patients, with most stopping due to ICI-induced T1DM.

### Clinical response

At median follow-up of 21.9 months (range 8.4-82.4 months), no patients in the melanoma group had progressive disease or died from their cancer. One patient on active treatment for stage IV disease died as a likely complication of his T1DM, but had no evidence of cancer progression prior to this unfortunate event. For the 4 patients who received active treatment, the best responses were 2 complete response (CR) and 2 partial response (PR). The 6 patients who received adjuvant therapy had no evidence of disease at follow-up (median follow-up 35.9 months). Median progression-free survival and overall survival were not reached in these patients. Seven of 8 patients with other cancers received active treatment, and 1 received neoadjuvant treatment. In the active treatment group, the best responses were 1 PR, 2 stable disease (SD) and 4 with progression of disease. The patient who received neoadjuvant therapy had a complete pathologic response. At data cut off, 3 were deceased, 3 had progression of disease and 1 did not. Median PFS and OS were 2.7 months and 18.6 months, respectively.

### Biomarkers and immunophenotypic data

PEA analysis of the 2 melanoma patients who developed ICI-induced T1DM versus 5 controls who did not develop any irAE was illustrated in the heat map (**Figure 2**). This is at the baseline prior to starting immunotherapy to show differences between the cohorts. Red illustrates higher expression while blue illustrates relatively lower expression in patients who developed ICI induced T1DM. While not statistically significant given the small sample size, there were visualized differences between groups. Lower levels of inflammatory pathway proteins (IL-6 and OSMR (oncostatin M regulator), present in IL-6 signaling) were found in the patients that developed ICI-induced T1DM. Furthermore, proteins related to metabolism were found to be increased in the ICI-induced T1DM patients. These included GLO1 (glyoxalase I), which is involved in the pyruvate pathway and glutathione reduction, and DXCR (dicarbonyl and L-xylulose reductase), which catalyzes diacetyl reductase and L-xylulose reductase reactions that play a role in glucose metabolism. ICI-induced T1DM patients also saw increased levels of certain proteins involved in cell signaling and communication. These included NPTN (neuroplastin), an Ig family transporter protein involved in cell-to-cell interactions, and ENPP5 (ectonucleotide pyrophosphatase/phosphodiesterase family member 5), a transmembrane glycoprotein that functions in neuronal cell communication.

## Discussion

To our knowledge, this is the largest reported case series describing oncologic outcomes, clinical attributes, and proteomic data from patients that developed ICI-induced T1DM at a single institution. While melanoma patients had excellent outcomes (100% ORR), in other solid tumors, outcomes were mixed, with most patients having progression of disease. All patients received anti-PD-1 therapy (nivolumab, cemiplimab or pembrolizumab) either as sole immunotherapy or, for 3 patients, as nivolumab in combination with ipilimumab. This is consistent with prior literature documenting anti-PD-1 therapy as opposed to anti-CTLA-4 (ipilimumab) therapy having a stronger predilection for disease (13). The mechanism underlying this observation is complex, but prior animal studies have shown that the PD-1/PD-L1 axis is more important to the self-tolerance of pancreatic beta cells than CTLA-4, given the low rates of ICI-induced T1DM from ipilimumab (14).

Our case series had important clinical implications. Patients began to show significantly elevated glucose levels 3 weeks prior to T1DM onset compared to pre-ICI baseline **(** **Figure 3** **).** This is a unique finding not reported in prior studies of predictive biomarkers, or prior case series (2, 5–12). Of note, glucose levels were not significantly elevated 6 weeks prior to presentation. This finding, taken in conjunction with some patients presenting in DKA with A1c <6.5%, suggests that the progression of pancreatic beta cell destruction and resulting glucose intolerance is very rapid, with development of the disease in a short period of time, in the span of days to weeks. Any significant increase in glucose on routine testing should prompt further evaluation or, at minimum, increased monitoring for ICI-induced T1DM. Early diagnosis is the only way to prevent further complications such as DKA. Through duration of follow-up, all patients required insulin therapy, highlighting the known irreversibility of this irAE (15). Interestingly, despite a lack of contraindication to resuming therapy, most patients did not receive further ICI therapy after developing T1DM. It is important for clinicians to recognize that ICI-induced T1DM should not preclude patients from further ICI therapy if indicated.

There are several proposed distinct subtypes of ICI-induced T1DM (16). Fulminant T1DM, characterized by rapid onset of T1DM presenting with DKA, usually presents with normal or near normal A1c (16). Two of our patients with A1c <7 may represent this subtype. Another proposed subtype is a phenotype like “decompensated” type 2 diabetes, which presents with a high A1c and detectable C-peptide, but new insulin dependency with or without prior history of type 2 diabetes. Five of our patients presenting with high A1c and low C-peptide levels were consistent with this group. One would expect positive autoantibody prevalence reflecting the proposed mechanism, with a more frequent prevalence in the fulminant variety, and less prevalent in a phenotype like type 2 diabetic decompensation. Another proposed cause is secondary to prior ICI induced pancreatitis, which is possible in our patient with an elevated lipase. Few patients had a lipase level checked, and it would be helpful in future workup. In our case series, only 3 of the 10 tested patients had positive autoantibodies which is similar prevalence to other published cohorts (30% vs 40-50%). It might be explained that most of the subtypes we observed were more like decompensated T2DM rather than a fulminant T1DM which could be present in slightly higher abundance in other studies (2, 6–12). Prior cohorts have suggested some correlation between the number of positive autoantibodies and shortened time from treatment to onset of T1DM (10). The three patients in our cohort did not support any clear conclusions, having time to onset comparable to our average.

Thirteen of 18 patients (72%) experienced at least 1 other irAE, with most experiencing multiple irAEs. Endocrinopathies are relatively common in patients with ICI-induced T1DM. One systematic review found ICI-induced thyroid dysfunction in 24% of cases (2). Eight in our cohort (44%) had other endocrine irAEs, comparable to published literature. It is not clear if prior severe irAEs are a predictor of future severe irAEs (T1DM). Only three of 18 patients had developed a severe (grade 3 or higher) irAE prior to their diagnosis of T1DM. Severe irAEs have been previously cited in one large case series as a poor prognostic factor, as patients are not often able to resume their ICI therapy (17). In our cohort, this was not the case, as none of these patients had progression of disease.

While limited by small sample size, our PEA analysis suggested several interesting relative differences. Decreased levels of proteins involved in inflammatory pathways such as IL-6, OSMR, and AMBP in patients who developed ICI-induced T1DM could help support the excellent response. IL-6 and OSMR play a role in tumor proliferation by activating transcription factors. Therapies targeting these pathways have been shown to be inhibitory to tumor growth (18). The IL-6 signaling pathway has also been described as promoting self-tolerance of pancreatic beta cells in autoimmune T1DM, although this is not well understood (19). Increased expression of proteins in metabolic pathways like GLO1 and DXCR were seen in T1DM patients without clear implications. GLO1 has been shown in several preclinical models to promote tumor cell proliferation, and one study showed GLO1 upregulating PD-L1 expression, suggesting positive treatment response when it is targeted (20, 21). Finally, increased levels of cell signaling proteins such as NPTN and ENPP5 were also noted. ENPP5 has been cited as a biomarker for increased insulin resistance in non-insulin dependent diabetes (22). NPTN is described as an important factor in T-cell activation, helping explain excellent response (23). This interpretation is entirely speculative and subject to confounding factors between patients but could be a clue for future larger studies with subject matching that is required for validation.

Despite ICI-induced T1DM, patients with melanoma displayed excellent clinical response and survival. No patient in this group experienced either progression of disease in the active treatment group or recurrence in the adjuvant group. In comparison, the other malignancies group only had two on active therapy that did not progress. Prior case series note a favorable response in melanoma compared to around 50% response rates to anti-PD-1 therapy in melanoma in general (24). More specifically immunotherapy trials involving patients with advanced (stage III or IV), unresectable disease noted a PFS over a comparable time period in the 40-50% range (25, 26). While patients with lower recurrence stage IIIA or IIIB disease need longer follow up times to determine clinical outcome, the 5 patients with metastatic disease all responded and none have relapsed with a mean follow up time of 39 months. Reasons for this excellent clinical response are difficult to determine although proteomic data may help guide future research. While there is a known association between severe irAEs and good clinical outcomes, in many instances this may be due to immortal time bias (27).

## Limitations

While our search criteria were broad, and our prevalence falls within the expected range, it is possible some patients with ICI-induced T1DM were not included. There was a patient identified during our EDW based screening who could not be included due to lack of documentation of lab testing needed to validate the diagnosis as ICI-induced T1DM. Other patients could have been missed by our search entirely, as our institution is a referral center for one of the country’s largest geographic catchments, and many patients were more likely to be hospitalized acutely closer to home. This also highlights the necessity of further diagnostic testing when patients present with possible irAEs or suspicious history. Further diagnostic testing of great interest would be HLA typing in these patients, which was not available in our study. Our case series is biased heavily towards melanoma, and it is difficult to see trends with other malignancies. While the response in melanoma patients was excellent, it is limited by the small size of the cohort. The follow up time for our patients with stage IIIA or IIIB melanoma may not be long enough to see a difference given the long PFS known for these stages.

## Conclusion

As a point of future study, germline, HLA, and immunophenotypic data could be helpful in providing insight and predictive value in determining which patients are at higher risk of ICI-induced T1DM. Clinicians should be aware of the rapid onset and irreversibility of ICI-induced T1DM, with evidence of elevated blood glucose 3 weeks prior to diagnosis. Positive treatment outcomes in patients with melanoma who develop ICI-induced T1DM may help guide treatment considerations. Larger studies are needed to elucidate strategies to identify high-risk patients and improve treatment outcomes for this rare irAE.

## Data availability statement

The original contributions presented in the study are included in the article/supplementary materials. Further inquiries can be directed to the corresponding author.

## Ethics statement

The studies involving human participants were reviewed and approved by 00138167. The patients/participants provided their written informed consent to participate in this study.

## Author contributions

JM and JPM identified cases. JM and SH-L verified and analyzed patient clinical data. SH-L and MK analyzed proteomic data. JM and JPM drafted the manuscript. All authors contributed to the article and approved the submitted version.
